# Thiamine May Be Beneficial for Patients With Ventilator-Associated Pneumonia in the Intensive Care Unit: A Retrospective Study Based on the MIMIC-IV Database

**DOI:** 10.3389/fphar.2022.898566

**Published:** 2022-06-23

**Authors:** Luming Zhang, Shaojin Li, Xuehao Lu, Yu Liu, Yinlong Ren, Tao Huang, Jun Lyu, Haiyan Yin

**Affiliations:** ^1^ Department of Intensive Care Unit, The First Affiliated Hospital of Jinan University, Guangzhou, China; ^2^ Department of Clinical Research, The First Affiliated Hospital of Jinan University, Guangzhou, China; ^3^ Department of Orthopaedics, The First Affiliated Hospital of Jinan University, Guangzhou, China; ^4^ Guangdong Provincial Key Laboratory of Traditional Chinese Medicine Informatization, Guangzhou, China

**Keywords:** ICU, ventilator-associated pneumonia, thiamine, IPW, mortality

## Abstract

**Background:** Ventilator-associated pneumonia (VAP) is a common infection complication in intensive care units (ICU). It not only prolongs mechanical ventilation and ICU and hospital stays, but also increases medical costs and increases the mortality risk of patients. Although many studies have found that thiamine supplementation in critically ill patients may improve prognoses, there is still no research or evidence that thiamine supplementation is beneficial for patients with VAP. The purpose of this study was to determine the association between thiamine and the prognoses of patients with VAP.

**Methods:** This study retrospectively collected all patients with VAP in the ICU from the Medical Information Mart for Intensive Care-IV database. The outcomes were ICU and in-hospital mortality. Patients were divided into the no-thiamine and thiamine groups depending upon whether or not they had received supplementation. Associations between thiamine and the outcomes were tested using Kaplan-Meier (KM) survival curves and Cox proportional-hazards regression models. The statistical methods of propensity-score matching (PSM) and inverse probability weighting (IPW) based on the XGBoost model were also applied to ensure the robustness of our findings.

**Results:** The study finally included 1,654 patients with VAP, comprising 1,151 and 503 in the no-thiamine and thiamine groups, respectively. The KM survival curves indicated that the survival probability differed significantly between the two groups. After multivariate COX regression adjusted for confounding factors, the hazard ratio (95% confidence interval) values for ICU and in-hospital mortality in the thiamine group were 0.57 (0.37, 0.88) and 0.64 (0.45, 0.92), respectively. Moreover, the results of the PSM and IPW analyses were consistent with the original population.

**Conclusion:** Thiamine supplementation may reduce ICU and in-hospital mortality in patients with VAP in the ICU. Thiamine is an inexpensive and safe drug, and so further clinical trials should be conducted to provide more-solid evidence on whether it improves the prognosis of patients with VAP.

## Introduction

Ventilator-associated pneumonia (VAP) refers to a lung parenchyma infection that occurs in patients with artificial airways (tracheal intubation or tracheotomy) who receive invasive mechanical ventilation (IMV) for at least 48 h (Kalil et al., 2016). With the maturity of modern rescue technology, ventilators and invasive diagnosis and treatment techniques have been widely used. IMV is one of the main cornerstones of life support in intensive care units (ICUs). This increases the VAP incidence, which studies have found to range from 5 to 40% in patients receiving IMV(Čiginskienė et al., 2019; Papazian et al., 2020). Once VAP occurs, it often causes weaning difficulties, thereby prolonging ICU stays and hospitalization times, increasing the costs for the patient, or even endangering their life and causing death (American Thoracic Society; Infectious Diseases Society of America, 2005; Zimlichman et al., 2013). Epidemiology suggests that the VAP-related all-cause mortality rate in critically ill patients can be as high as 50% (Papazian et al., 2020). The timely diagnosis and treatment of VAP is therefore of great significance. The main treatment for VAP is currently antibiotics (Metersky and Kalil, 2018), but primary disease treatment, prevention and treatment of risk factors leading to VAP, nutritional support, immunotherapy, and enhanced nursing can improve VAP prognoses (Modi and Kovacs, 2020; Ścisło et al., 2022).

Thiamine (also called vitamin B1) is a water-soluble vitamin, and its biologically active form is thiamine pyrophosphate in cells, which is an essential coenzyme in the tricarboxylic acid cycle during glucose metabolism and participates in human energy production (Polegato et al., 2019). Thiamine can also maintain the redox state of cells and participate in the antioxidant pathway by producing reduced nicotinamide adenine dinucleotide phosphate (NADPH) and glutathione (Mallat et al., 2016). In addition to these traditional functions, recent studies have found that thiamine derivatives also have some nonenzymatic functions, such as involvement in gene expression, stress response, and neural signal transduction regulation (Tylicki and Siemieniuk, 2011; Aleshin et al., 2019). These important roles of thiamine form the basis of thiamine supplementation in critically ill patients, and it has been explored in many trials in intensive care environments. For example, thiamine is often studied in the form of a drug combination with hydrocortisone and ascorbic acid (so-called HAT therapy) (Iglesias et al., 2020). Several studies have found that HAT therapy is associated with organ dysfunction improvement, decreased Sequential Organ Failure Assessment scores, increased lactate clearance, and reduced mortality in patients with sepsis (Marik, 2018; Woolum et al., 2018). Other than in patients with sepsis, a retrospective cohort found that HAT therapy is also associated with a reduced in-hospital mortality risk in patients with severe pneumonia (Kim et al., 2018). Thomas et al. found beneficial effects from thiamine, vitamin C, and vitamin D in patients with COVID-19, acute respiratory distress syndrome, and sepsis (Jovic et al., 2020). Thiamine is a particularly safe and inexpensive treatment, but warrants larger clinical trials to provide more evidence for its utility. At present, there is no research or evidence to show whether thiamine supplementation can improve the prognosis of patients with VAP. This study therefore aimed to determine the effect of thiamine on the prognosis of patients with VAP based on the Medical Information Mart for Intensive Care (MIMIC)-IV database, which will provide more evidence for clinical thiamine application and thus help to improve prognoses.

## Methods

### Data Source and Population

MIMIC is a large, single-center, freely available database developed by the Massachusetts Institute of Technology (Yang et al., 2020; Wu et al., 2021). Several versions have been released, and the latest version, MIMIC-IV (version 1.0), was released on 16 March 2021. This version contains comprehensive information on more than 200,000 patients hospitalized between 2008 and 2019, and it uses a modular approach to organize its data structure and highlight data sources to better utilize different data sources (Goldberger et al., 2000). The database was approved by the Massachusetts Institute of Technology (Cambridge, Mass.) and the Beth Israel Deaconess Medical Center (Boston, Mass.), and consent was obtained for collection of the original data (Johnson et al., 2021). Patients in the database were anonymized, so informed consent was not required. The data in this database can be accessed and extracted after researchers have completed the appropriate coursework and obtained the associated certificate.

All patients with a diagnosis of VAP in the ICU were included in this study on the basis of International Statistical Classification of Diseases, 9th and 10th Revisions (ICD-9 and ICD-10). If patients were admitted to ICU more than once, only data on their first admission was selected. Patients who stayed in the ICU or hospital for less than 24 h and who were younger than 18 years were excluded. Patients with VAP were divided into the no-thiamine and thiamine groups depending on whether or not they had received supplementation (including via intravenous and oral routes).

### Data Extraction

Patient information was extracted from the database using Structured Query Language. Demographic information included age, sex, Body Mass Index [BMI = weight (kg)/height^2^(m)], and ethnicity. Patient clinical information included admission type, first care unit, Acute Physiology Score III (APSIII), and interventional therapy [vasopressor use, continuous renal replacement therapy (CRRT) use]. The main comorbidities of patients included sepsis, myocardial infarction, congestive heart failure, hypertension, cerebrovascular disease, chronic pulmonary disease, liver disease, renal disease, diabetes, and malignant cancer. Laboratory test indicators were collected from the first record after admission to the ICU, vital signs were the worst values on the first day of the ICU, and urine output was the total on the first day of the ICU.The outcomes of this study were ICU and in-hospital mortality.

### Statistical Analyses

Testing revealed that the continuous variables in this study did not conform to a normal distribution, and so they are expressed as median and interquartile (IQR) values, and differences between the two groups were determined using Mann-Whitney U tests. Categorical variables are presented as numbers and percentages, and differences between groups were determined using chi-square and Fisher’s exact tests.

Kaplan-Meier (KM) curves and log-rank tests were used to assess whether thiamine supplementation influenced patient survival. We then constructed two Cox proportional-hazards models to analyze how thiamine affected the outcomes. No covariates were adjusted for in model I. While in model II, patients’ general characteristics, disease severity scores, interventions, comorbidities, laboratory findings and vital signs were adjusted in order to balance the impact of these factors on patient outcomes. Variance inflation factors (VIFs) were used to test for multicollinearity among the independent variables before performing multivariate COX regression.

To guarantee the robustness of the findings, we used propensity-score matching (PSM) and inverse probability weighting (IPW) to reduce the baseline differences between the two groups. When performing PSM and IPW analyses, the presence of missing values was not allowed. Therefore, the multiple imputation function in the “mice” package of our R software was applied to fill in the missing covariate values beforehand. The propensity scores for patients in the thiamine group were estimated using a multivariate logistic regression model followed by one-to-one nearest-neighbor matching with a 0.05 caliper width. XGBoost (Extreme Gradient Boosting) is an efficient gradient boosting decision tree algorithm, which can be used in the R software package “twang” to estimate relevant propensity scores (Yuan et al., 2020). We incorporated 43 covariates into the XGBoost model, obtained estimated propensity scores as weights, and finally used the IPW model to generate weighted cohorts (McCaffrey et al., 2013). This process introduced two new groups: the PSM and weighted populations. Then, similar to for the original population, univariate and multivariate Cox regression analyses were applied to these two populations to obtain a doubly robust estimation.

We also analyzed the effect of thiamine on the prognoses of different patient subgroups. Subgroups included age (<65 and ≥65 years), sex (male and female), and all of the comorbidities listed above. The interactions between the subgroups were further analyzed.

A two-tailed probability value of *p* < 0.05 was considered statistically significant. All statistical analyses in this study were performed using R software (version 4.1.0).

## Results

### Baseline Characteristics

**FIGURE 1:**
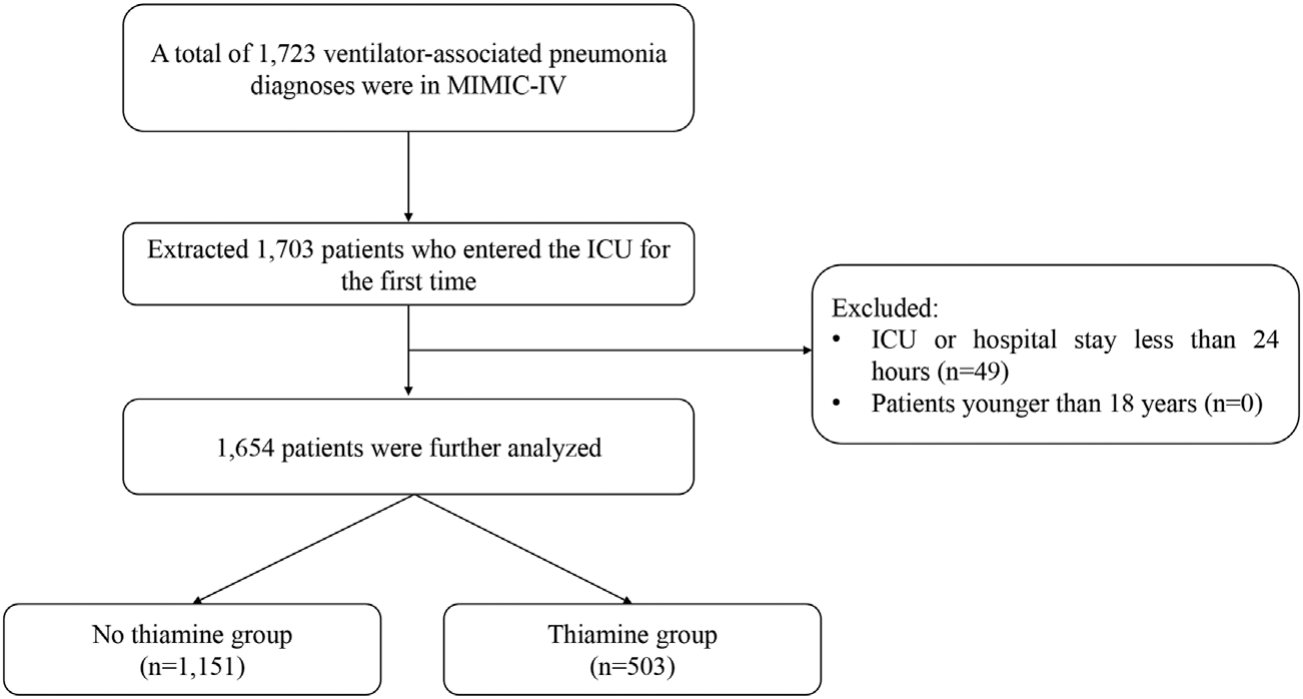
Inclusion and exclusion flowchart of the study.

The study ultimately finally included 1,654 patients with VAP, comprising 1,151 in the no-thiamine group and 503 in the thiamine group (Figure 1). Patients in the no-thiamine group were older than those in the thiamine group (median [IQR] = 66.00 [55.00, 77.00] years old vs. 61.00 [49.00, 70.00] years old); larger proportions of patients in both groups were male (61.7 and 65.6%, respectively); patients in the thiamine group had higher APSIII scores than those in the no-thiamine group (median [IQR] = 66.00 [50.00, 86.00] vs. 71.00 [51.00, 92.00]); patients hospitalized due to an emergency in the two groups accounted for 61.0 and 53.9% of those in the no-thiamine and thiamine groups, respectively; and sepsis accounted for the highest proportion of comorbidities in both groups (94.2 and 96.2%, respectively). More baseline characteristic information is provided in Table 1.

**TABLE 1:** Baseline characteristics of the original population.

	No Thiamine Group	Thiamine Group	*p*-Value	Missing data (%)
	1,151	503		
Age	66.00 (55.00, 77.00)	61.00 (49.00, 70.00)	<0.001	0
Gender (%)			0.143	0
male	710 (61.7)	330 (65.6)		
female	441 (38.3)	173 (34.4)		
BMI	28.31 (24.47, 34.17)	27.99 (23.68, 33.67)	0.365	0
APSIII	66.00 (50.00, 86.00)	71.00 (51.00, 92.00)	0.003	0
Ethnicity (%)			0.049	0
White	690 (59.9)	275 (54.7)		
Black	128 (11.1)	52 (10.3)		
others	333 (28.9)	176 (35.0)		
Admission type (%)			0.008	0
emergency	702 (61.0)	271 (53.9)		
others	449 (39.0)	232 (46.1)		
First careunit (%)			0.298	0
MICU/SICU	861 (74.8)	389 (77.3)		
others	290 (25.2)	114 (22.7)		
Vasopressor (%)			0.079	0
no	756 (65.7)	307 (61.0)		
yes	395 (34.3)	196 (39.0)		
CRRT (%)			0.002	0
no	1,128 (98.0)	478 (95.0)		
yes	23 (2.0)	25 (5.0)		
Duration of IMV(hour)	124.00 (56.00, 241.00)	169.00 (74.00, 310.00)	<0.001	0
Comorbidities				
Sepsis (%)			0.109	0
no	67 (5.8)	19 (3.8)		
yes	1,084 (94.2)	484 (96.2)		
Myocardial infarct (%)			0.283	0
no	934 (81.1)	420 (83.5)		
yes	217 (18.9)	83 (16.5)		
Congestive heart failure (%)			0.020	0
no	750 (65.2)	358 (71.2)		
yes	401 (34.8)	145 (28.8)		
Hypertension (%)			0.013	0
no	636 (55.3)	244 (48.5)		
yes	515 (44.7)	259 (51.5)		
Cerebrovascular disease (%)			0.004	0
no	835 (72.5)	399 (79.3)		
yes	316 (27.5)	104 (20.7)		
Chronic pulmonary disease (%)			0.184	0
no	808 (70.2)	370 (73.6)		
yes	343 (29.8)	133 (26.4)		
Liver disease (%)			<0.001	0
no	1,014 (88.1)	341 (67.8)		
yes	137 (11.9)	162 (32.2)		
Renal disease (%)			0.015	0
no	874 (75.9)	410 (81.5)		
yes	277 (24.1)	93 (18.5)		
Diabetes (%)			0.047	0
no	777 (67.5)	365 (72.6)		
yes	374 (32.5)	138 (27.4)		
Malignant cancer (%)			0.287	0
no	1,029 (89.4)	459 (91.3)		
yes	122 (10.6)	44 (8.7)		
Vital signs				
mMAP (mmhg)	58.00 (51.00, 65.00)	59.00 (52.00, 66.00)	0.111	0.06
mHR (-min)	69.00 (60.00, 82.00)	74.00 (61.00, 86.00)	<0.001	0.06
mRR (-min)	13.00 (10.00, 15.38)	13.00 (10.00, 16.00)	0.966	0.12
mSpO2 (%)	93.00 (90.00, 96.00)	93.00 (90.00, 95.00)	0.218	0.06
mT (°C)	37.61 (37.17, 38.22)	37.64 (37.11, 38.22)	0.735	5.08
Laboratory tests				
WBC(k/uL)	12.00 (8.80, 16.25)	11.60 (8.20, 16.40)	0.174	0.36
Lymphocytes (%)	9.00 (5.30, 14.40)	9.20 (5.97, 14.62)	0.504	0.36
Neutrophils (%)	81.90 (74.88, 87.00)	79.95 (72.07, 85.40)	0.001	0.36
Hemoglobin (g/dl)	10.50 (8.90, 12.30)	10.70 (8.88, 12.50)	0.525	0.36
Platelet (k/uL)	198.00 (144.00, 265.50)	181.00 (112.00, 245.00)	<0.001	0.36
RDW (%)	14.60 (13.60, 16.10)	14.70 (13.60, 16.80)	0.151	0.48
AG(mEq/L)	14.00 (12.00, 17.00)	15.00 (13.00, 19.00)	<0.001	0
Lactate (mmol/L)	1.60 (1.10, 2.60)	1.80 (1.20, 3.20)	0.004	4.53
PaCO2(mmhg)	42.00 (36.00, 50.00)	41.00 (34.00, 49.00)	0.018	1.69
PaO2(mmhg)	109.00 (71.00, 200.50)	101.00 (59.50, 182.00)	0.006	1.69
INR	1.20 (1.10, 1.50)	1.30 (1.10, 1.60)	0.212	2.06
PTT(s)	30.60 (26.90, 37.88)	31.10 (27.30, 38.10)	0.532	2.06
AST (IU/L)	39.00 (25.00, 78.00)	53.00 (29.00, 125.25)	<0.001	8.65
ALT (IU/L)	29.00 (17.00, 56.75)	31.00 (18.00, 66.50)	0.024	9.25
Bilirubin, total (mg/dL)	0.60 (0.40, 1.00)	0.80 (0.40, 1.80)	<0.001	9.61
Albumin (g/dl)	2.90 (2.50, 3.40)	2.90 (2.50, 3.30)	0.235	16.51
Glucose (mg/dl)	138.00 (111.00, 179.00)	136.00 (108.00, 178.50)	0.163	0
Creatinine (mg/dL)	1.00 (0.70, 1.50)	1.00 (0.70, 1.90)	0.199	0
Urine output (ml)	1,590.00 (962.50, 2350.00)	1,371.50 (757.50, 2223.75)	<0.001	2.72

### Survival Analysis and Cox Proportional-Hazards Regression Model

**FIGURE 2:**
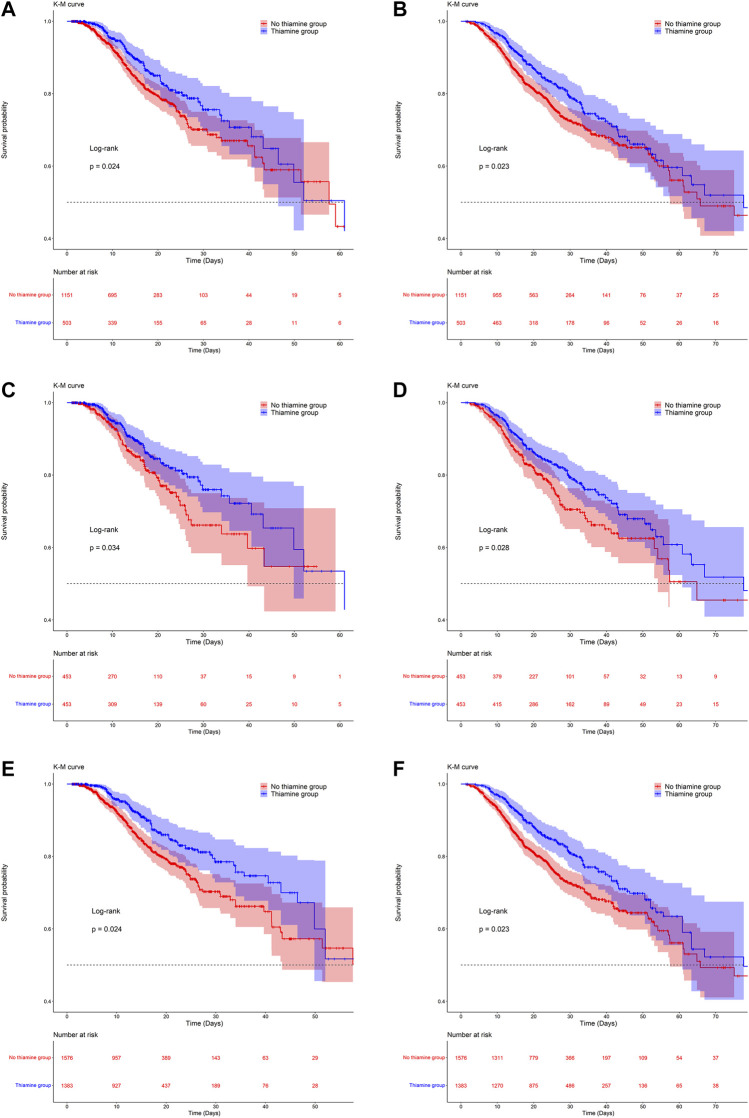
Kaplan-Meier survival curves between groups. a, c and e are the ICU mortality risk for the original population, the PSM population and the IPW population; b, d, and f are the in-hospital mortality risk for the original population, the PSM population and the IPW population.

ICU mortality rates were 15.6 and 13.9% in the no-thiamine and thiamine groups, respectively, and the corresponding in-hospital mortality rates were 22.6 and 21.7%. The KM survival curves indicated that the survival probability differed significantly between the two groups. Patients with VAP who received thiamine had significantly higher survival odds in both the ICU and in-hospital (Figure 2).

Supplementary Table S1 listed the VIFs of each covariate, they were all less than 4, indicating that there was no multicollinearity between the variables. The Cox proportional-hazards model results are listed in Table 2. The hazard ratios (HRs) were less than 1 in the thiamine group when compared with the no-thiamine group in both the unadjusted model and the model adjusted for all confounders; that is, patients in the thiamine group had lower ICU and in-hospital mortality risks than did those in the no-thiamine group. After adjusting for the covariates mentioned above as confounding factors, the HR (95% confidence interval [CI]) values for ICU and in-hospital mortality in the thiamine group were 0.57 (0.37, 0.88) and 0.64 (0.45, 0.92), respectively, indicating that the ICU and in-hospital mortality risks were 0.57 and 0.64 times higher than those in the no-thiamine group, respectively (Table 2).

**TABLE 2:** Results of Cox proportional hazard models.

	Model I		Model II	
Outcomes	HR (95%CI)	*p*-Value	HR (95%CI)	*p*-Value
Original population				
ICU Mortality				
Thiamine				
no	Reference		Reference	
yes	0.73 (0.55,0.96)	0.025	0.57 (0.37,0.88)	0.011
In-hospital Mortality				
Thiamine				
no	Reference		Reference	
yes	0.77 (0.61,0.96)	0.023	0.64 (0.45,0.92)	0.015
After PSM				
ICU Mortality				
Thiamine				
no	Reference		Reference	
yes	0.68 (0.49,0.97)	0.035	0.62 (0.42,0.91)	0.015
In-hospital Mortality				
Thiamine				
no	Reference		Reference	
yes	0.73 (0.56,0.96)	0.028	0.72 (0.53,0.98)	0.036
After IPW				
ICU Mortality				
Thiamine				
no	Reference		Reference	
yes	0.64 (0.48,0.85)	0.002	0.65 (0.48,0.89)	0.007
In-hospital Mortality				
Thiamine				
no	Reference		Reference	
yes	0.69 (0.54,0.87)	0.002	0.75 (0.57,0.97)	0.029

Abbreviations: HR, hazard ratio; CI, confidence interval.

Models were derived from Cox proportional hazards regression models.

Model I was not adjusted for covariates.

Model II covariates were adjusted for age, sex, BMI, race, admission type, first care unit, APSIII, vasopressor use, CRRT, sepsis, myocardial infarction, congestive heart failure, hypertension, cerebrovascular disease, chronic pulmonary disease, liver disease, renal disease, diabetes, malignant cancer, WBC, neutrophils, lymphocytes, hemoglobin, RDW, platelets, AG, PaCO2, PaO2, lactate, creatinine, AST, ALT, total bilirubin, albumin, INR, PTT, glucose, mHR, mMAP, mRR, mT, mSpO2, and urine output.

### Propensity-Score Matching and Inverse Probability of Treatment Weighting

After PSM and IPW, baseline differences between the two groups improved substantially, but there were still differences in a few variables (Supplementary Figure S1; Supplementary Table S2). The KM survival curves of the matched and weighted populations indicated a trend consistent with that for the original population (Figure 2). As with the original population, we also applied univariate and multivariate Cox regression analyses to the matched and weighted populations. After the multivariate Cox regression, the HRs (95% CI) for ICU and in-hospital mortality in the thiamine group were 0.62 (0.42, 0.91) and 0.72 (0.53, 0.98), respectively, in the PSM population, and 0.65 (0.48, 0.89) and 0.75 (0.57, 0.97) in the weighted population (Table 2).

**FIGURE 3:**
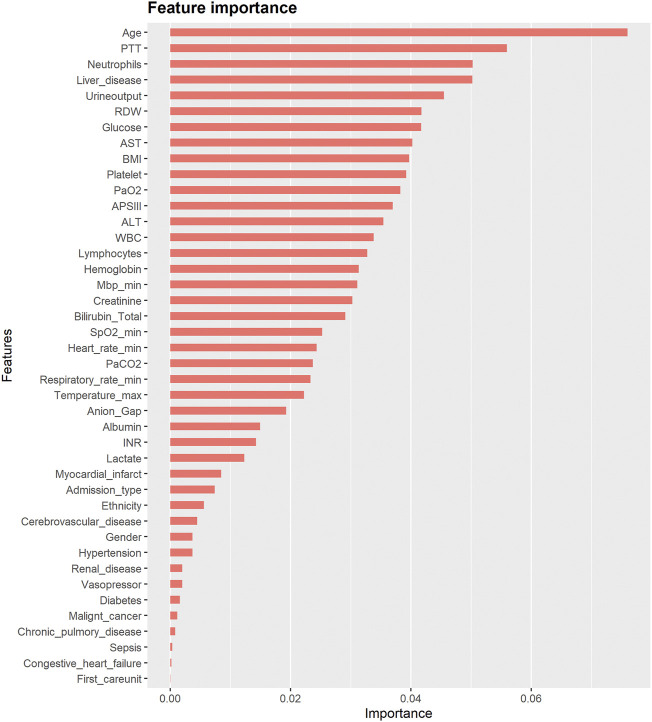
The contribution of each covariate to the XGBoost model.

Figure 3 also shows rankings of contributions to the propensity score of 43 covariates in the XGBoost model, which reflected the degree of influence of different covariates on the groups or the degree of imbalance between groups. The figure shows that the five highest-ranked variables in order were age, PTT, neutrophils, liver disease, and urine output.

### Subgroup Analysis

The results of the subgroup analysis are listed in Table 3, and there was no significant interaction between the thiamine and no-thiamine groups in each stratified population.

**TABLE 3:** Subgroup analysis of relationship between groups and mortality.

	ICU Mortality			In-hospital Mortality		
	HR (95%CI)	*p*-value	p-interaction	HR (95%CI)	*p*-value	p-interaction
Age			0.887			0.865
<65 (*n* = 831)	0.84 (0.39,1.84)	0.667		0.79 (0.44,1.43)	0.440	
≥65 (*n* = 823)	0.50 (0.27,0.88)	0.021		0.64 (0.39,1.04)	0.073	
Gender			0.405			0.072
male (*n* = 1,040)	0.57 (0.31,1.03)	0.064		0.69 (0.44,1.09)	0.115	
female (*n* = 614)	0.500 (0.23,1.11)	0.088		0.47 (0.24,0.92)	0.029	
Sepsis						
no (*n* = 86)	(NA)			(NA)		
yes (*n* = 1,568)	0.59 (0.37,0.90)	0.015		0.67 (0.47,0.95)	0.024	
Myocardial infarct			0.649			0.476
no (*n* = 1,354)	0.56 (0.34,0.93)	0.023		0.68 (0.47,1.00)	0.051	
yes (*n* = 300)	0.45 (0.16,1.27)	0.131		0.53 (0.21,1.36)	0.186	
Congestive heart failure			0.654			0.894
no (*n* = 1,108)	0.53 (0.28,0.98)	0.044		0.66 (0.41,1.07)	0.089	
yes (*n* = 546)	0.59 (0.28,1.26)	0.171		0.58 (0.32,1.08)	0.171	
Hypertension			0.836			0.520
no (*n* = 880)	0.68 (0.36,1.26)	0.219		0.77 (0.47,1.27)	0.300	
yes (*n* = 774)	0.39 (0.19,0.80)	0.010		0.54 (0.31,0.94)	0.029	
Cerebrovascular disease			0.175			0.455
no (*n* = 1,234)	0.58 (0.35,0.95)	0.032		0.68 (0.45,1.01)	0.059	
yes (*n* = 420)	0.55 (0.13,2.25)	0.403		0.52 (0.18,1.47)	0.218	
Chronic pulmonary disease			0.277			0.074
no (*n* = 1,178)	0.62 (0.36,1.07)	0.086		0.74 (0.49,1.11)	0.152	
yes (*n* = 476)	0.32 (0.13,0.76)	0.010		0.35 (0.17,0.75)	0.006	
Liver disease			0.685			0.596
no (*n* = 1,355)	0.63 (0.38,1.05)	0.075		0.68 (0.44,1.03)	0.074	
yes (*n* = 299)	0.21 (0.05,0.82)	0.024		0.44 (0.10,0.95)	0.038	
Renal disease			0.797			0.854
no (*n* = 1,284)	0.57 (0.33,0.97)	0.039		0.66 (0.44,0.99)	0.047	
yes (*n* = 370)	0.44 (0.16,1.21)	0.112		0.39 (0.16,0.95)	0.037	
Diabetes			0.719			0.822
no (*n* = 1,142)	0.58 (0.34,1.00)	0.051		0.62 (0.41,0.96)	0.032	
yes (n = 512)	0.61 (0.26,1.45)	0.267		0.63 (0.31,1.26)	0.181	
Malignant cancer						
no (*n* = 1,488)	0.55 (0.34,0.90)	0.166		0.63 (0.42,0.94)	0.027	
yes (*n* = 166)	(NA)			(NA)		

Hazard ratio (95% CI): from Cox proportional hazards regression models. The covariate adjustment was consistent with Model II, in Table 2.

## Discussion

Based on the MIMIC-IV database, this study was the first to determine the effects of thiamine supplementation on ICU and in-hospital mortality risks among patients with VAP. The results were very gratifying, confirming that thiamine—as an inexpensive, easily available, and relatively safe drug—is related to improving the prognosis of patients with VAP, which was also verified using PSM and IPW. The results were stable and reliable, providing a new basis for clinical VAP treatment research.

Many published studies have assessed the relationship between thiamine and patients with sepsis, or have conducted clinical studies involving combined HAT therapy. A subset of studies did not exhibit beneficial effects of thiamine, such as one multicenter randomized clinical study indicating that HAT therapy did not provide faster relief of septic shock compared with intravenous hydrocortisone alone, but this study did not evaluate the possible individual effects of vitamin C and thiamine separately (Fujii et al., 2020). There are also some studies with similar results to ours that thiamine was strongly associated with improved organ dysfunction and reduced mortality in patients with sepsis (Donnino et al., 2010; Woolum et al., 2018). With the recent spread of COVID-19, the role of thiamine in this disease is gradually being explored (Jovic et al., 2020). A two-center, noninterventional, retrospective study found that the thiamine group had significantly lower 30-days mortality for critically ill patients admitted to the ICU with confirmed COVID-19 (Al Sulaiman et al., 2021).

Thiamine cannot be synthesized endogenously and so can only be obtained from food (Frank, 2015). However, patients with VAP in ICUs are often in a state of fasting or eating less due to their critical condition, resulting in insufficient thiamine intake (Attaluri et al., 2018). As a key coenzyme in glycolysis, thiamine plays a key regulatory role in the process of mitochondrial ATP synthesis to provide cells with energy, and so the lack of thiamine will inevitably affect mitochondrial function (Belsky et al., 2018). Impaired mitochondrial function can lead to cell dysfunction, leading to the dysfunction or even failure of various organs (Singer et al., 2004). Extensive published studies have found that thiamine deficiency is a common phenomenon in ICUs(Manzanares and Hardy, 2011; van Snippenburg et al., 2017), and can lead to serious complications in critically ill patients with heart failure, neuropathy, gastrointestinal dysfunction, and lactic acidosis (Katta et al., 2016; Attaluri et al., 2018; Woolum et al., 2018). Thiamine supplementation therefore helps to restore mitochondrial function and reduce the likelihood of organ dysfunction occurrence, thereby improving patient prognoses. In addition to playing an important role in energy metabolism, the production of the major components of redox reactions in the body are all inextricably linked to thiamine (Mallat et al., 2016). In patients with VAP, cell structure changes due to the inflammatory response and tissue hypoxia, and the balance of oxidation and antioxidant systems is dysfunctional, resulting in excessive levels of oxidative stress products such as reactive oxygen species (Galley, 2011). Experiments have confirmed that the thiamine level is positively correlated with glutathione peroxidase activity (Depeint et al., 2006), which is the main component of the cellular antioxidant system and has a strong scavenging effect on oxygen free radicals (Cominetti et al., 2011).

In summary, exogenous thiamine supplementation was observed to not only contribute to the energy recovery of patients with VAP, reducing the occurrence of some complications, but also relieve the state of oxidative stress and play an anti-inflammatory role, which is of great significance in improving patient prognosis and survival. Regarding drug safety, a clinical study of patients with Wernicke’s encephalopathy found that there were no obvious side effects from thiamine treatment, even after high-dose oral administration at 500 mg three times a day, indicating that thiamine is relatively safe in clinical applications (Donnino et al., 2007).

### Strengths and Limitations

To the best of our knowledge, this was the first study to investigate the relationship between thiamine and the prognosis of patients with VAP. Various statistical methods were used to ensure the stability of the results. The large number of population samples in the MIMIC-IV database also provided a solid foundation for our research. Of course, this study also had some limitations. Firstly, this study had a retrospective design. When identifying the study population, we determined the diagnosis of VAP by ICD codes in the MIMIC-IV database. However, we could not avoid this problem due to the possible interobserver variability in the diagnosis of VAP (Klompas, 2010). In future studies, focus on patients with ventilator-associated events may have a higher clinical applicability. Secondly, although we tried our best to balance confounding factors, there were still some potential confounding biases. Thirdly, our study only focused on whether patients with VAP had received thiamine supplementation, and so specific and optimal thiamine doses need to be explored in future prospective studies. Moreover, there is no routine test for measuring thiamine levels, and so no further studies were performed on the thiamine levels of patients in this study.

## Conclusion

Thiamine supplementation may reduce ICU and in-hospital mortality in patients with VAP in the ICU. Thiamine is an inexpensive and safe drug, and so further clinical trials should be conducted to provide more-solid evidence on whether it improves the prognosis of patients with VAP.

## Data Availability

Publicly available datasets were analyzed in this study. This data can be found here: The data were available on the MIMIC-IV website at https://mimic.physionet.org/, https://doi.org/10.13026/a3wn-hq05.

## Abbreviations

VAP: ventilator-associated pneumonia; ICU: intensive care unit; KM: Kaplan-Meier; PSM: propensity-score matching; IPW: inverse probability weighting; IMV: invasive mechanical ventilation; HAT therapy: hydrocortisone, ascorbic acid and thiamine; MIMIC: Medical Information Mart for Intensive Care; ICD: International Statistical Classification of Disease; BMI: Body Mass Index; APSIII: Acute Physiology Score III; CRRT: continuous renal replacement therapy; WBC: white blood cells; RDW: red blood cell distribution width; AG: anion gap; AST: aspartate aminotransferase; ALT: alanine aminotransferase; INR: international normalized ratio; PTT: partial thromboplastin time; mHR: min heart rate; mMAP: min value of mean arterial pressure; mRR: min respiratory rate; mT: max temperature; mSpO2: min SpO2; IQR: interquartile; VIF: variance inflation factors; XGBoost: Extreme Gradient Boosting; HR: hazard ratios.
