# Supplementary figures and images for: Thiamine May Be Beneficial for Patients With Ventilator-Associated Pneumonia in the Intensive Care Unit: A Retrospective Study Based on the MIMIC-IV Database
Source: Front Pharmacol. 2022 Jun 23;13:898566. doi: 10.3389/fphar.2022.898566 (PMC9259950; doi:10.3389/fphar.2022.898566)

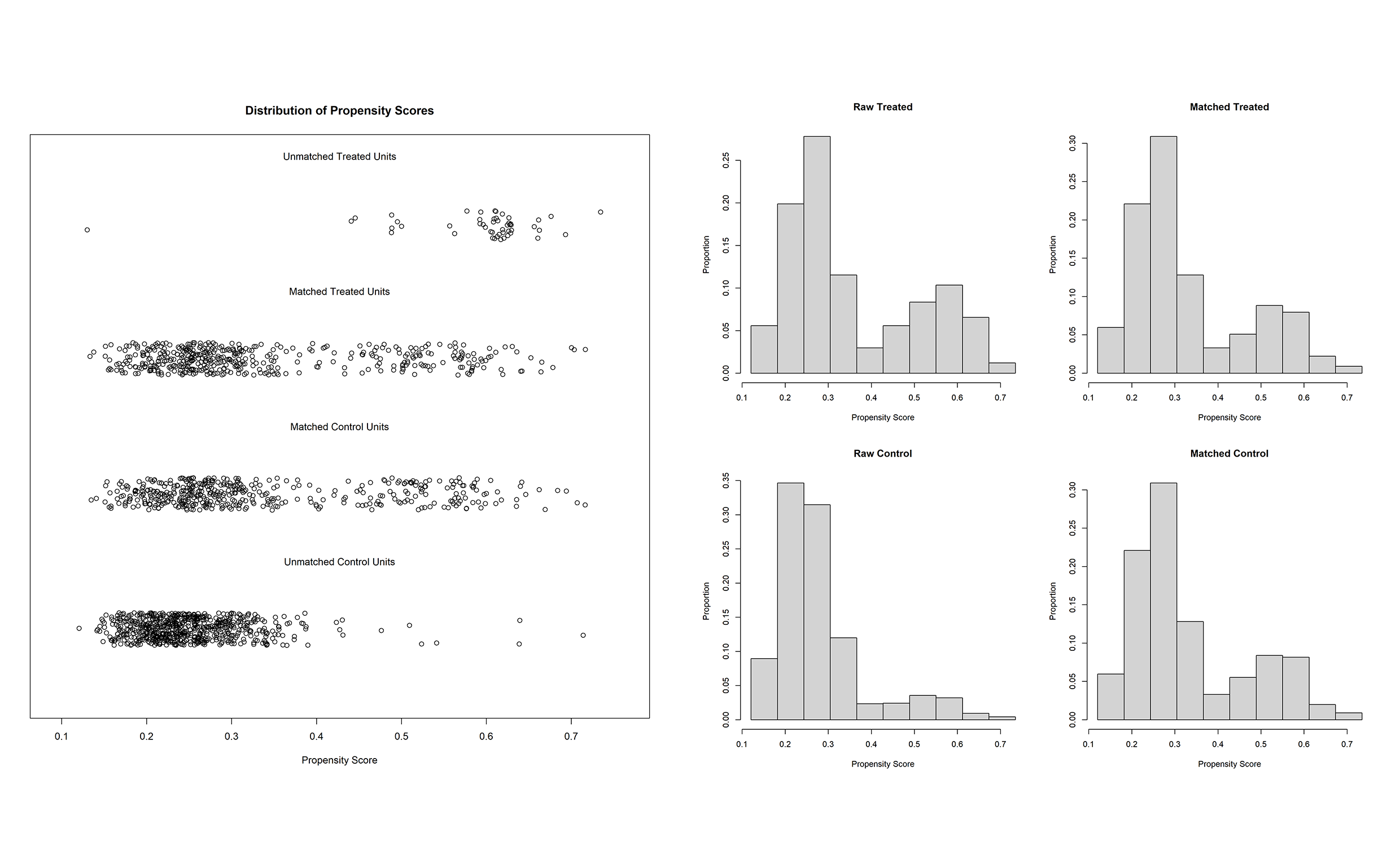

Supplement: Supplementary file 2 [file Image1.TIF]
